# Minutes of Missouri State Dental Society

**Published:** 1866-05

**Authors:** H. Judd

**Affiliations:** St. Louis


### MINUTES OF MISSOURI STATE DENTAL SOCIETY.

St. Louis, Oct. 31st, 1865.

Pursuant to a call made by the St. Louis Dental Society, the Dentists of the State convened at New Church Hall, corner of Sixth and St. Charles Streets, at 10 o'clock, A. M.

On motion of Dr. Peebles, Dr. John S. Clark was called to the chair, as temporary Chairman, and Dr. G. S. Morse as Secretary.

On motion, a committee, consisting of Drs. Forbes, Sloan, Comstock, Depp, and Eames, were appointed by the Chair to draft a constitution and present the same to the meeting.

The convention took a short recess, when the house was called to order by the Chairman.

Dr. Peebles proposed the following query: Why is it that rubber does not vulcanize to the same extent, at all times, with the same amount of heat and length of time?

The subject was discussed at considerable length by Drs. Jones, Blake, Payne, McCoy, Peebles, Hovey, Morse, and others; after which the committee, appointed for that purpose, reported a constitution, which was read, and on motion, unanimously adopted.

A committee of five, composed of Drs. Peebles, Samuels, Tindall, Blake, and McCoy, were appointed to nominate permanent officers.

On motion, adjourned to half-past 2 o'clock.

Association met pursuant to adjournment. Committee reported names for officers, as follows: for President, H. J. McKellops; for 1st Vice-President, G. S. Morse; for 2nd Vice-President, M. McCoy; for Recording Secretary, H. Judd; for Corresponding Secretary, J. Payne; and for Treasurer, A. M. Leslie,—who were duly elected.

The Chairman appointed Drs. Leslie and Forbes to conduct the President, elect, to the chair.

After some very appropriate remarks by the President, on motion of Dr. Forbes, the Nominating Committee were requested to nominate an Executive Committee of three, and Drs. Blake, Sloan and Samuels were nominated and duly elected.

On motion, Dr. Leslie was requested to furnish a synopsis of the proceedings of the Association, for publication in the Dental Journals. After a short discussion upon the use of arsenic for killing nerves, the Association took a recess to allow members to sign the constitution. and thirty-six members subscribed their names thereto. Association again came to order and resumed the discussion. After a spirited discussion of some length, by different members, the Association adjourned to half-past 7 o'clock.

Association met at half-past 7 o'clock, and was called to order by Dr. Morse, Vice-President. The subject of exposed pulp was taken up and discussed at some length, after which there followed a discussion upon vulcanized rubber.

The Executive Committee reported, as a subject for discussion, Periostitis. After some remarks by Dr. Samuels, on motion of Dr. Leslie, it was ordered that to-morrow forenoon be set apart for clinics.

On motion of Dr. Peebles, the Executive Committee were empowered to fill the vacancy in said committee, occasioned by the absence of Dr. Blake. Committee appointed Dr. Peebles to fill said vacancy.

The subject of Periodentitis was resumed and participated in by Drs. Eames, Judd, and others, after which the following resolution was offered by Dr. Payne:

*Resolved*, That it is the sense of this Association that no no man is justified in taking a student for a less time than two years, and then only when such student pledges himself to graduate at a Dental College before engaging in the practice of dentistry.

After remarks by Drs. Sloan, Forbes, Clark, and Payne, followed by an able speech from Dr. Peebles, the resolution was unanimously adopted.

The Executive Committee appointed Drs. Forbes, McKellops, Peebles, Payne, Eames, and Barron, to operate at the clinic in the presence of the other members of the Association. Adjourned to 2 o'clock, to-morrow.

Association met at 2 o'clock, P. M.; President in the chair; minutes of last meeting were read and approved.

On motion, Drs. McCuen, Payne, and Jones were appointed a committee to report upon the subject of vulcanized rubber, at the next annual meeting of the Association.

On motion of Dr. Blake, Dr. Spyer was requested to read a paper on "The effects of Disease on the Teeth." The essay having been read—on motion of Dr. Peebles, Dr. Spyer was requested to furnish a copy for publication, with the proceedings of the Association.

Letters were read from Drs. J. A. Price, of Weston, and J. F Hassell, of Lexington; when, on motion, Drs. Leslie, Hovey, and Read were appointed a committee to nominate delegates to the American Dental Association. A recess followed to allow members time to examine instruments presented by Dr. McKellops and others.

Upon coming to order, it was agreed, on motion of Dr. Morse, that when the Association finally adjourns it shall do so to meet at St. Louis, on the first Tuesday of June next. On motion, adjourned to half-past 7 o'clock, P. M.

Association met pursuant to adjournment. Minutes of last meeting were read and approved.

Dr. McCoy introduced, as a subject of consideration, cases of extreme absorption of lower jaw, and enquired the best method of retaining, under such circumstances, a lower piece in situ.

After remarks by Drs. McKellops, Payne, Hovey, Spyer, and McCuen, the regular subject for discussion, "fang filling," was resumed and discussed by various members. The following resolution was offered by Dr. Clark, and was unanimously adopted:

*Resolved*, That the thanks of this Association are due, and are hereby tendered to Dr. H. E. Peebles, for his constant labors to effect a full organization of the Dentists of Missouri into a State Society; he having commenced this labor as early as July, 1864, by extensive correspondence and consultation with members of the profession.

On motion of Dr. Forbes, it was ordered that a suitable preamble be prefixed to the resolution, and that it be written in a plain hand, and placed in a neat gilt frame, and signed by the President and Recording Secretary, and presented to Dr. Peebles as a testimonial of the appreciation which the members of the Association have for these services.

The Nominating Committee presented the following report: Your committee, in nominating delegates to the American Dental Association, would respectfully propose the appointment of the following gentlemen:

J. K. Stark, Independence, Mo.; Edward Hale, Jr., St. Louis, Mo.; T. W. Reed, Macon, Mo.; J. S. Clark, St. Louis, Mo.; E. Hovey, Springfield, Mo.; E. McCuen, Lauinana, Mo.; Geo. Crawford, St. Louis, Mo.; G. W. Tindall, Kansas City, Mo.; H. Judd, St. Louis, Mo.

A. M. Leslie,
T. W. Reed,
E. Hovey,
*Committee.*

The report of the committee was received, and the gentlemen named in the report duly elected as delegates to the American Dental Association.

On motion of Dr. Forbes, the subject for discussion at the evening session be "Why have we failed to preserve teeth." On motion, adjourned to half-past 7 o'clock.

Association met at half-past 7 o'clock. President in chair. Discussion of considerable length upon the subject presented by Dr. Forbes; when, on motion, it was resolved, that the Executive Committee be requested to lay out a series of subjects for discussion at the next annual meeting of the Association, and place the same in the hands of the Corresponding Secretary to be printed, and copies to be mailed to each member of the Association.

On motion of Dr. Forbes, the Executive Committee was authorized to draw on the Treasurer for funds to defray the expenses of the meeting.

President appointed Dr. Park a delegate to the American Dental Convention.

On motion of Dr. Morrison, a subscription was ordered to be opened for the *Dental Register*; after which, the following resolution was adopted by Dr. Hovey:

*Resolved*, That the thanks of this Association are due, and are hereby tendered to Drs. Forbes, McKellops, Peebles,

Payne, Eames, and Barron, for their courteous consideration and gentlemanly bearing towards the members of the Association, in kindly opening their offices and tendering their services, not only in the clinical exhibition thus afforded us, but also in their readiness to impart any and all professional information at their command.

On motion, the above resolution was unanimously adopted.

On motion, Association adjourned to meet in St. Louis, the first Tuesday in June, 1866.

H. JUDD, *Rec. Sec'y.*

